# Subarachnoid Hemorrhage is Followed by Pituitary Gland Volume Loss: A Volumetric MRI Observational Study

**DOI:** 10.1007/s12028-019-00764-x

**Published:** 2019-06-20

**Authors:** Verena Rass, Elisabeth Schoenherr, Bogdan-Andrei Ianosi, Anna Lindner, Mario Kofler, Alois J. Schiefecker, Lukas Lenhart, Max Gaasch, Marie-Theres Pertl, Christian F. Freyschlag, Bettina Pfausler, Margarete Delazer, Ronny Beer, Claudius Thomé, Astrid Ellen Grams, Christoph Scherfler, Raimund Helbok

**Affiliations:** 1 Department of Neurology, Neurocritical Care Unit, Medical University of Innsbruck, Anichstrasse 35, 6020 Innsbruck, Austria; 2 Department of Radiology, Medical University of Innsbruck, Anichstrasse 35, 6020 Innsbruck, Austria; 3 Institute of Medical Informatics, UMIT: University for Health Sciences, Medical Informatics and Technology, Eduard Wallnoefer-Zentrum 1, 6060 Hall, Austria; 4 Department of Neuroradiology, Medical University of Innsbruck, Anichstrasse 35, 6020 Innsbruck, Austria; 5 Department of Neurosurgery, Medical University of Innsbruck, Anichstrasse 35, 6020 Innsbruck, Austria

**Keywords:** Subarachnoid hemorrhage, Pituitary gland volume, Cerebral aneurysm, Critical care, Neurology, Neuropsychological outcome

## Abstract

**Background:**

Subarachnoid hemorrhage (SAH) is a devastating disease associated with high mortality and morbidity. Besides neurological sequelae, neuropsychological deficits largely contribute to patients’ long-term quality of life. Little is known about the pituitary gland volume (PGV) after SAH compared to healthy referents and the association of PGV with long-term outcome including cognitive function.

**Methods:**

Sixty consecutive non-traumatic SAH patients admitted to the neurological intensive care unit between 2010 and 2014 were enrolled. 3-Tesla magnetic resonance imagining was performed at baseline (16 days) and 12 months after SAH to measure PGV semi-automatically using the software iPlan Net 3.5.0. PGV was compared to age and sex matched healthy referents. The difference between baseline and 1-year-PGV was classified as increase (> 20 mm^3^ PGV increase), stable (± 20 mm^3^), or decrease (> 20 mm^3^ PGV decrease). In addition, total intracerebral volume was calculated. Neuropsychological testing was applied in 43 SAH patients at 1-year follow up encompassing several domains (executive, attention, memory) and self-assessment (questionnaire for self-perceived deficits in attention [German: FEDA]) of distractibility in mental processes, fatigue and decrease in motivation. Multivariable regression with multivariable generalized linear models was used for comparison of PGVs and for subgroup analysis to evaluate a potential association between PGV and neuropsychological outcome.

**Results:**

Patients were 53 years old (IQR = 44–63) and presented with a median Hunt&Hess grade of 2 (IQR = 1–3). SAH patients had a significantly lower PGV both at baseline (360 ± 19 mm^3^, *p* < 0.001) and 1 year (367 ± 18 mm^3^*p* < 0.001) as compared to matched referents (mean 505 ± 18 mm^3^). PGV decreased by 75 ± 8 mm^3^ in 28 patients, increased by 120 ± 22 mm^3^ in 22 patients and remained stable in 10 patients at 1-year follow-up. PGV in patients with PGV increase at 12 months was not different to healthy referents (*p* = 0.062). Low baseline PGV was associated with impaired executive functions at 1 year (adjOR = 8.81, 95%-CI = 1.46–53.10, *p* = 0.018) and PGV decrease within 1 year was associated with self-perceived worse motivation (FEDA; Wald-statistic = 6.6, *df* = 1, *p* = 0.010).

**Conclusions:**

Our data indicate significantly lower PGVs following SAH. The association of sustained PGV decrease with impaired neuropsychological long-term outcome warrants further investigations including neuroendocrine hormone measurements.

## Introduction

Subarachnoid hemorrhage (SAH) is a devastating disease still being associated with a high mortality and morbidity. Despite improved survival rates due to advances in the individualized neurocritical care management [1], quality of live is strongly diminished in a substantial number of SAH survivors [2]. Although many patients are classified as having a favorable functional outcome, neuropsychological and cognitive deficits are common. This includes decreased executive functions and memory impairments, as well as emotional deficits such as depression, anxiety and fatigue [2]. While it is well known that the bleeding itself and hospital complications substantially contribute to these deficits [2, 3], early identification of patients at high risk remains difficult. Some of the typical neuropsychological deficits after SAH resemble clinical symptoms of hypopituitarism [4], which is commonly observed in the acute and subacute phase after SAH [5–8]. Potential pathophysiological mechanisms resulting in endocrine dysfunction include direct compression of the hypothalamic-pituitary complex by the aneurysm itself, intracranial hypertension, compromised perfusion during initial circulatory arrest after SAH with subsequent ischemic injury, hydrocephalus, and iatrogenic causes through pharmacological treatment or procedure related injuries [5, 9].

Pituitary gland volume (PGV) assessed in the subacute phase after SAH may be an adequate radiological biomarker to identify patients at high risk for subsequent deficits resembling endocrine dysfunctions. To the best of our knowledge there are no studies on PGV after SAH. Following traumatic brain injury (TBI) PGV increased soon after and normalized within 1 year to a volume comparable to healthy age and sex matched referents [10]. Moreover, pituitary imaging abnormalities were related to hypopituitarism after TBI [11]. In line with this observation, hypopituitarism in SAH patients was linked to hypothalamic magnetic resonance imaging (MRI) lesions [12]. Several radiographic parameters including global cerebral edema, left-sided infarction [3, 13] and focal lesions [14] have been associated with impaired neuropsychological test performance following SAH. In long-term, regional and global brain atrophy have been correlated with cognitive deficits after SAH [15–19].

In the current study we hypothesized, that atrophy of the pituitary gland occurs after SAH and may be associated with neuropsychological impairments. Therefore, we aimed (1) to quantify the PGV at baseline and at 1-year follow-up in comparison with healthy referents, (2) to assess disease related and demographic factors associated with decreased PGV after SAH and (3) to associate PGV in the subacute and chronic phase with neuropsychological outcome parameters.

## Methods

### Study Design, Setting and Participants

The study design was guided by the STROBE statement on observational cohort studies. We screened 183 consecutive patients with non-traumatic SAH admitted to the neurological intensive care unit (ICU) of a tertiary hospital (Medical University of Innsbruck) between April 2010 and December 2014. Out of these, 79 patients met the following inclusion criteria: (1) age greater or equal to 18 years, (2) ICU stay for more than 24 h, (3) baseline and 1-year follow-up MRI-scan, (4) exclusion of arteriovenous-malformation. Further radiological exclusion criteria encompassed (1) patients with macroadenoma (*N* = 1), (2) artifacts not compatible with volumetric PGV analysis (*N* = 6) and (3) missing sequences for volumetric PGV quantification (*N* = 12) leaving 60 patients eligible for final analysis of this observational study. Approval for this study was granted by the local ethics committee (Medical University of Innsbruck, AM4091-292/4.6). All patients gave informed consent according to local regulations in accordance with the Declaration of Helsinki.

### Clinical Management

Clinical management of patients after SAH conformed to international guidelines [20, 21]. Ruptured aneurysms were occluded by either clipping or coiling. Oral or intravenous nimodipine was administered prophylactically in all patients. All patients were followed for vasospasm with repetitive transcranial color coded duplex sonography (TCD, LOGIQ S8, GE Healthcare, Chicago, Illinois, United States). Vasospasm was defined as an elevation of mean velocities > 120 cm/s in the middle or anterior cerebral artery or a daily change in mean TCD-velocities > 50 cm/s. Severe vasospasm (> 200 cm/s) was further confirmed by catheter cerebral angiogram and nimodipine was administered intraarterial. Delayed cerebral ischemia was defined as clinical deterioration (decrease of ≥ 2 points on the Glasgow Coma Scale), occurrence of a new focal neurological deficit or a new infarction on computed tomography or MRI-scan not attributable to other causes [22]. The treatment of intracranial hypertension included osmotherapy with mannitol (15%) or hypertonic saline (10%) infusions. fT3, fT4 and TSH levels were routinely measured at admission and further during ICU stay following clinical necessity. Cortisol levels were measured in all patients with refractory hypotension or high need of catecholaminergic support. Patients’ baseline characteristics, complications and outcomes were prospectively collected in weekly meetings held by the study team and treating neurointensivists.

### MRI Data Acquisition and Image Postprocessing for Volumetric Analysis

MRI-scans of the brain were performed on a 3-Tesla whole-body MRI scanner (Magnetom Verio, Siemens Erlangen, Germany) using a 12-channel head coil at baseline (16 days) and 12 months after SAH. The MRI protocol was identical in all patients and included whole-brain T1-weighted, and fluid- attenuated inversion recovery, T2- and proton density-weighted. MRI sequences for coronal T1-weighted 3D magnetization prepared rapid gradient echo were: repetition time (TR) = 1800 ms; echo time (TE) = 2.18 ms; inversion time, TI = 900 ms; slice thickness: 1.2 mm; matrix: 384 × 512; number of excitations: 1; flip angel = 9°; field of view 220 × 165 mm.

**Fig. 1:**
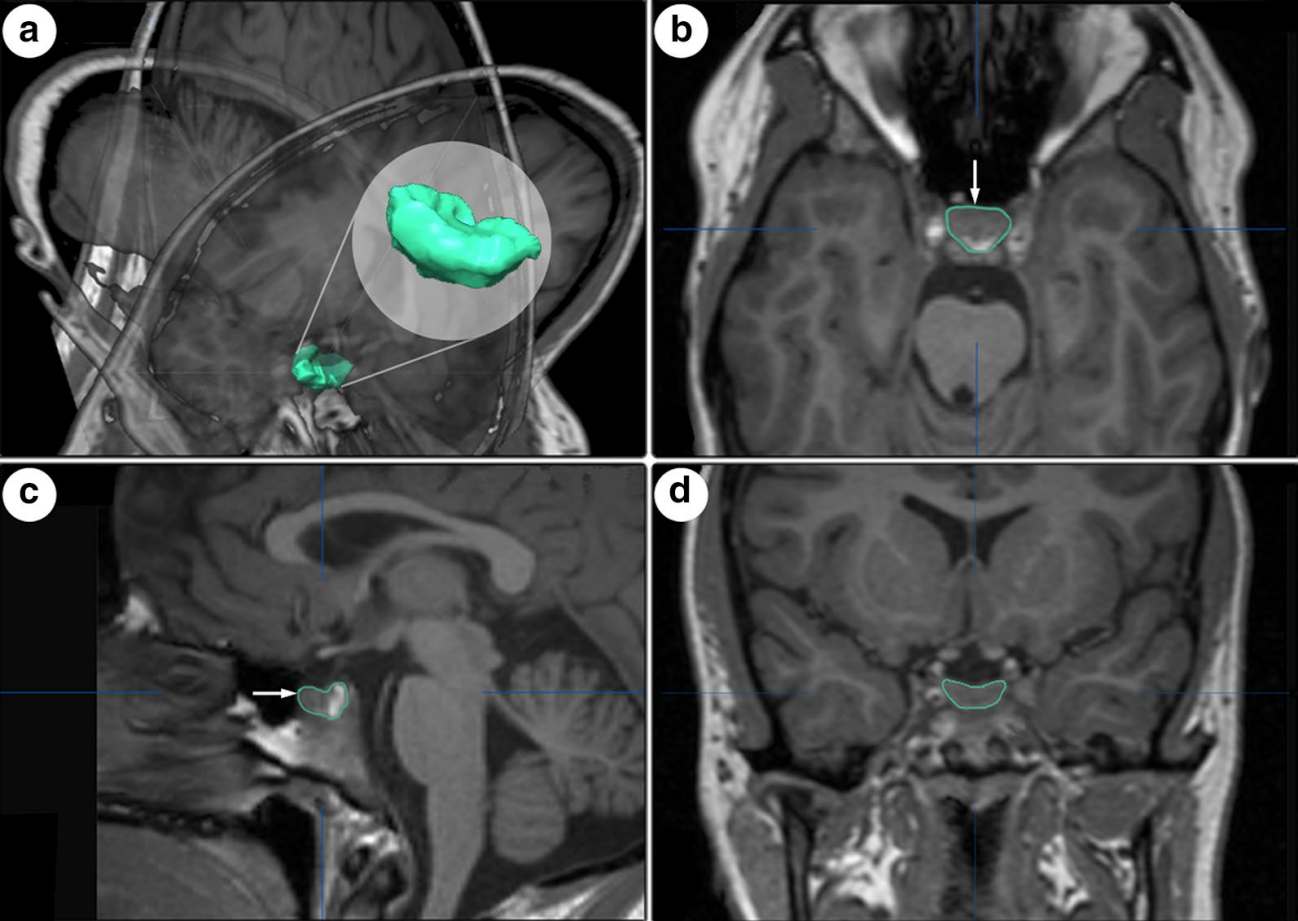
**a** Semiautomatic pituitary volumetry with the software iPlan Net showing 3 planes: **b** transverse, **c** sagittal and **d** coronal

PGVs were semi-automatically measured using the software iPlan Net 3.5.0 (Brainlab AG, Germany; Fig. 1) by ES. Coronal T1-weighted whole-brain sequences were selected, and the pituitary gland brushed on each slice before starting the segmentation. Pituitary volumes were obtained in cubic millimeters. Total intracranial volume (TIV) was calculated by segmenting and summing up the gray and white matter as well as cerebrospinal fluid compartments using the software package statistical parametric mapping (SPM12, Wellcome Department of Cognitive Neurology, London, UK) implemented in Matlab 9.2 (Mathsworks Inc., Sherborn, MA). To assess interrater reliability, the PGV of 20 patients was assessed by a second independent rater (AG), blinded to clinical data and the volumetric results.

Quantified volumes were compared to 60 age and sex matched healthy referents selected from the MRI database of the Department of Neuroradiology, Medical University of Innsbruck.

### Neuropsychological and Outcome Evaluation

Functional neurological outcome was assessed at 3 months by a study nurse blinded to the disease course of the patient and at 12 months by a medical doctor of the study team and rated with the modified Rankin Scale score (mRS). Unfavorable outcome was defined as mRS ≥ 3.

Additionally, neuropsychological in-person assessment was applied in 49 patients at 12 months after ictus, 43 completed all tests relevant for the current analysis. Several cognitive domains (executive, attention, orientation, verbal and visual memory, language and visual-spatial skills) were screened using a standardized test battery. Furthermore, questionnaires for symptoms of anxiety and depression [23] as well as the German Questionnaire for self-perceived deficits in attention, or FEDA (including 3 domains: distractibility in mental processes, fatigue and decrease in motivation) [24] were used. Screening of global cognitive functions was done by the Mini-Mental State Examination [25]. Several executive functions were evaluated by validated tests, including semantic verbal fluency (number of named animals within 60 s) by the Regensburger Verbal Fluency Test [26], verbal working memory by the Wechsler Memory Scale revised, digit span forwards and backwards [27] and visual conceptualization (clock drawing) [28]. As a global screening instrument of executive functions, the Frontal Assessment Battery (FAB) [29, 30] was used, which consists of 6 sub-tests including conceptualization (find similarities between objects), lexical fluency (S-words produced within 60 s), motor programming (fist-palm-edge motor series), sensitivity to interference (conflicting instructions requiring opposite response to a signal), inhibitory control (go no-go paradigm) and environmental autonomy (suppression of manual prehension behavior). The test results were interpreted by experienced neuropsychologists.

### Statistical Analysis

Continuous variables were assessed for normality and reported as mean ± standard error of mean or median and interquartile range (IQR). Categorical variables were reported as count and proportions in each group.

In order to study the SAH specific effect on PGV, our analysis was based on the comparison between PGV of SAH patients and healthy referents using relative PGV. The ratio of PGV_SAH_/PGV_REF_ was median 0.69 at baseline and 0.76 at follow-up. PGV was therefore dichotomized as low (< 0.69, < 0.76, respectively) and high PGV. Groups (low vs. high PGV, and PGV_SAH_/TIV vs. PGV_REF_/TIV at 1 year) were compared in univariate analysis using the *t* test, Mann–Whitney *U* test or *Fishers exact*-test, as appropriate. The difference between baseline and follow-up PGV was coded as increase (> 20 mm^3^ PGV increase), stable (± 20 mm^3^), or decrease (> 20 mm^3^ PGV decrease), based on the observation of a mean error of 21 mm^3^ (± 8) assessed in 3 control groups. Within-patient variations of PGV normalized to ∆TIV over 1 year were compared using the paired *t* test.

Multivariable generalized linear models were used to identify associations between relative PGVs (PGV_SAH_/PGV_REF_*100) or ∆PGV (PGV_FU_–PGV_baseline_ in SAH patients) and scores of neuropsychological tests including the FAB score (continuous variable) and FEDA questionnaire (continuous variable). For the multivariable model associating PGV with FAB, clinically important variables were entered along with potential confounders (education years, sex, age, H&H grade). All models were adjusted for TIV. Cases with missing values were included. All analyses and graphical representations were performed with SPSS (IBM SPSS Statistics, Version 24.0. Armonk, NY, USA). Statistical significance was attributed to a *p* value < 0.05.

## Results

### Baseline Characteristics

A total of 60 patients were included in final analysis. The median age was 53 (IQR = 44–63) years and 37 patients (62%) were women. The full spectrum of clinical severity grades of SAH patients was analyzed: twenty-one (35%) patients presented with an admission H&H grade of 1, 18 (30%) with 2, 9 (15%) with 3, 2 (3%) with 4 and 10 (17%) patients with a H&H grade of 5. Detailed information on admission variables and hospital complications is given in Table 1. Age (*p* = 0.972) and sex (*p* = 1.00) were not significantly different between SAH patients and healthy referents.

**Table 1:** Risk factors for low pituitary gland volume in 60 SAH patients relative to healthy referents

		Baseline			1-year follow-up		
		Low PGV^1^	High PGV	*P* value	Low PGV^1^	High PGV	*P* value
*Clinical characteristics*							
Age (years)	53 (44–63)	58 (49–66)	49 (40–60)	**0.011**	56 (46–63)	50 (41–64)	0.376
Female gender	37 (62)	20 (67)	17 (57)	0.596	18 (60)	19 (63)	1.0
Admission Hunt and Hess grade	2 (1–3)	2 (1–3)	2 (2–3)	0.173	2 (1–3)	2 (1–3)	0.288
Admission GCS	15 (8–15)	15 (10–15)	15 (3–15)	0.305	15 (10–15)	15 (4–15)	0.581
Hypertension history	24 (40)	16 (53)	8 (27)	0.064	13 (43)	11 (37)	0.792
Diabetes mellitus	2 (3)	2 (7)	0 (0)	0.492	2 (7)	0 (0)	0.492
Admission glucose in mg/dL	121 (109–149)	126 (110–162)	117 (109–145)	0.464	119 (109–152)	121 (110–150)	0.836
Loss of consciousness at ictus	14 (23)	6 (20)	8 (27)	0.761	6 (20)	8 (27)	0.761
*Admission radiological characteristics*							
Modified Fisher at admission	3 (2–4)	3 (2–4)	3 (2–4)	0.482	3 (3–4)	3 (2–4)	0.574
ICH on admission	9 (15)	3 (10)	6 (20)	0.472	5 (17)	4 (13)	0.731
Hydrocephalus requiring EVD	18 (30)	8 (27)	10 (33)	0.779	7 (23)	11 (37)	0.399
Aneurysm size	5 (3–8)	5 (3–8)	5 (3–9)	0.752	5 (3–8)	5 (3–9)	0.929
Aneurysm localization							
ACA, ACoA	25 (42)	14 (64)	11 (55)	0.754	14 (61)	11 (58)	1.00
ICA, MCA	9 (15)	3 (14)	6 (30)	0.269	4 (17)	5 (26)	0.707
Posterior circulation	8 (13)	5 (23)	3 (15)	0.700	5 (22)	3 (16)	0.709
Admission SEBES	1 (0–2)	1 (0–2)	1 (0–2)	0.815	1 (0–2)	1 (0–2)	0.512
*Brain compartments at baseline MRI*							
Total intracerebral volume in ccm	1409 ± 19	1376 ± 26	1443 ± 26	0.076	1408 ± 25	1434 ± 28	0.483
Gray matter in ccm	656 ± 10	639 ± 12	673 ± 17	0.107	633 ± 12	632 ± 19	0.966
White matter in ccm	416 ± 9	406 ± 10	426 ± 15	0.266	397 ± 10	403 ± 15	0.765
Cerebrospinal fluid in ccm	337 ± 13	330 ± 16	344 ± 20	0.608	377 ± 18	400 ± 23	0.709
*Aneurysm treatment*							
Coiling	28 (47)	17 (57)	11 (37)	0.192	16 (53)	12 (40)	0.748
Clipping	14 (23)	5 (17)	9 (30)	0.192	7 (23)	7 (23)	0.748
Non-aneurysmal SAH	18 (30)	8 (30)	10 (33)	0.779	7 (23)	11 (37)	0.399
*Hospital complications*							
Pneumonia	23 (38)	11 (37)	12 (40)	1.00	10 (33)	13 (43)	0.596
Ventriculitis	5 (8)	3 (10)	2 (7)	1.00	2 (7)	3 (10)	1.00
Urinary tract infection	16 (27)	9 (30)	7 (24)	0.771	7 (23)	9 (31)	0.567
Hyponatremia (< 130 mmol/L)^2^	12 (20)	5 (17)	7 (23)	0.748	7 (23)	5 (17)	0.748
Hypernatremia (> 150 mmol/L)^2^	7 (12)	3 (10)	4 (13)	1.00	4 (13)	3 (10)	1.00
Peak leukocytes^2^	14.2 (11.0–17.8)	14.6 (10.7–18.1)	13.9 (12.1–17.9)	0.841	14.5 (7.0–24.0)	13.9 (11.2–17.9)	0.950
Fever > 38.3 °C	31 (53)	14 (47)	17 (61)	0.306	14 (47)	17 (61)	0.306
Hydrocortison, cumulative dose in mg^2^	0 (0–623)	0 (0–161)	0 (0–826)	0.173	0 (0–161)	0 (0–826)	0.102
Vasospasm	29 (48)	16 (53)	13 (43)	0.606	14 (47)	15 (50)	1.00
Delayed cerebral ischemia	15 (25)	8 (27)	7 (23)	1.00	8 (27)	7 (23)	1.00
Intubated days	1 (0–12)	1 (0–9)	1 (0–16)	0.393	1 (0–10)	2 (0–13)	0.569
*Outcome characteristics*							
Length of ICU stay in days	16 (10–29)	15 (7–28)	16 (12–29)	0.554	14 (7–24)	16 (11–31)	0.357
Poor functional outcome at 3 months (mRS > 2)	19 (32)	8 (27)	11 (37)	0.580	7 (23)	12 (40)	0.267
Poor functional outcome at 12 months (mRS > 2)^3^	10 (20)	5 (21)	5 (19)	1.00	4 (16)	6 (24)	0.725

Significant differences between low and high PGV in univariate analysis (*P* < 0.05) is given in bold

Data are given in median (IQR), mean ± SEM or *N*(%)

*ACA* anterior cerebral artery, *ACoA* anterior communicating artery*, EVD* external ventricular drain, *GCS* Glasgow Coma Scale, *ICA* internal carotid artery, *ICH* intracerebral hemorrhage, *ICU* intensive care unit, *MCA* middle cerebral artery, *MRI* magnetic resonance imaging, *mRS* modified Rankin Scale, *PGV* pituitary gland volume, *SAH* subarachnoid hemorrhage, *SEBES* Subarachnoid hemorrhage Early Brain Edema Score

^1^Relative to PGV of age and sex matched healthy referents

^2^Within 15 days

^3^Available in 50 patients

MRI-scans were obtained at a median interval of 16 (IQR = 14–25) and 385 (IQR = 372–395) days after SAH. Nineteen patients (32%) had low cortisol levels together with a high catecholamine demand, prompting continuous hydrocortisone administration with a cumulative dose of 923 mg (IQR = 623–1308, assessed within the first 15 days of admission). Routinely measured levels of TSH, fT4 and fT3 in 55 patients revealed 3 patients (6%) with latent hyperthyroidism, 1 patient (2%) with manifest hypothyroidism, 9 (16%) with latent hypothyroidism and 42 (76%) with normal thyroid gland function during ICU stay. In 7 patients oral thyroxin was administered during ICU stay, whereas at 1-year FU 5 patients still required thyroxin.

### PGV and Its Dynamic Change in SAH Patients Compared to Healthy Referents

**Fig. 2:**
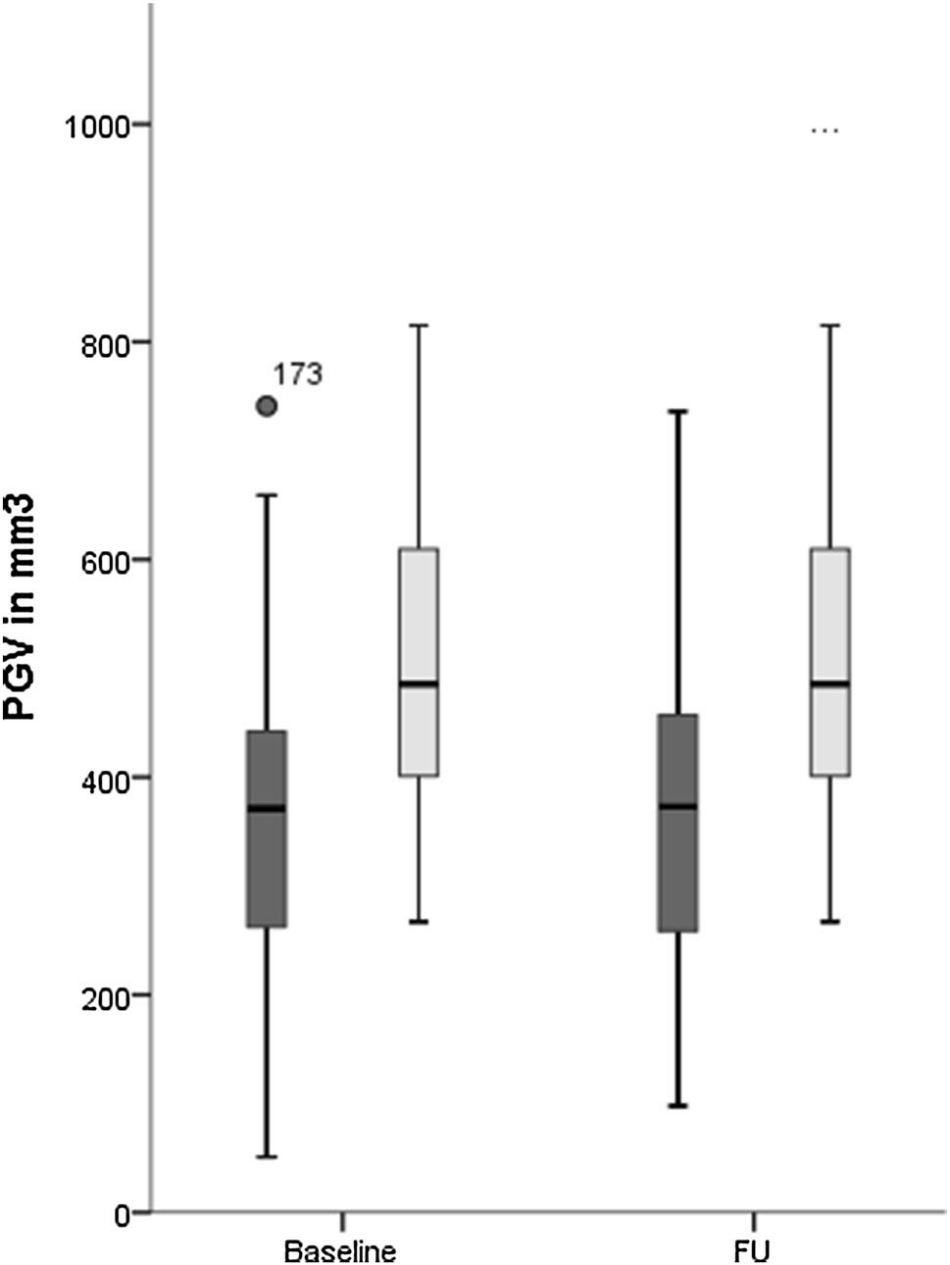
The box plots represent lower PGVs in SAH patients both at baseline (16 days) and 1-year follow-up (FU) in comparison to healthy referents. The central line shows the 50th percentile, the upper and lower lines the 75th and 25th percentile and the short horizontal bars at the ends the 90th and 10th percentiles. The darker shades of gray represent SAH patients while the lighter shades of gray represent healthy referents

Interobserver reliability of PGV was high with an intraclass correlation coefficient (ICC) of 0.979. PGV was significantly lower both at baseline (360 ± 19 mm^3^, *p* < 0.001) and 1-year follow-up (367 ± 18 mm^3^, *p* < 0.001) in SAH patients as compared to healthy age and sex matched referents (505 ± 18 mm^3^, Fig. 2). Similarly, the percentage of PGV from TIV (PGV/TIV*100) was lower in SAH patients at baseline (0.0252%, IQR = 0.0166–0.0315, *p* < 0.001) and follow-up (0.0251%, IQR = 0.0181–0.0335, *p* < 0.001) as compared to healthy referents (0.0332%, IQR = 0.0274–0.0421). The ratio of PGV_SAH_/PGV_REF_ was 0.69 at baseline and 0.76 at 12 months. Overall, PGV did not change over 1 year in SAH patients (*p* = 0.604). However in sub-analysis, significant dynamic changes of PGV over 1 year were identified: PGV decreased by 75 ± 8 mm^3^ in 28/60 patients (47%; 405 ± 27–329 ± 23 mm^3^, *p* < 0.001), increased by 120 ± 22 mm^3^ in 22 patients (37%; 298 ± 23–418 ± 36 mm^3^, *p* < 0.001) and remained stable in 10 patients (17%; 368 ± 37–360 ± 35 mm^3^, *p* = 0.153). PGV in patients with PGV increase at 12 months was not different to healthy referents (*p* = 0.062). ∆PGV over 1 year was neither associated with H&H grade (*p* = 0.251) nor with age (*p* = 0.091) or sex (*p* = 0.094). The PGV in all tests was relative to TIV.

### Risk Factors for Lower PGV at Baseline and Follow-Up in SAH Patients

Elderly SAH patients had significantly lower PGV at baseline (*p* = 0.011) relative to healthy referents. However, the analysis did not yield any significant association between disease relevant risk factors and relative low PGV at baseline and 1-year FU as detailed in Table 1.

### PGV and Neuropsychological Long-Term Outcome

Median scores and interquartile ranges of neuropsychological tasks and cognitive outcomes at 1 year are given in Table 2. Frequencies of patients scoring in impaired ranges were classified according to age-scaled norms or predefined age- and education-specific cutoff values.

**Table 2:** Neuropsychological test results at 1-year follow-up

	*N*^a^	Median (IQR)	*N*(%)		
			Average	Slightly impaired	Impaired
MMSE	49	29 (27–29)	38 (76)	NA	11 (22)
CLOX	47	12 (11–13)	37 (79)	NA	10 (21)
Semantic verbal fluency (RWT)	47	20 (16–27)	37 (79)	6 (13)	4 (9)
FAB score	47	16 (14–18)	34 (72)	1 (2)	12 (26)
Digit span forwards, test score (WMS-R)	48	6 (5–6)	32 (67)	16 (33)	–
Digit span backwards, test score (WMS-R)	48	4 (3–5)	34 (71)	13 (27)	1 (2)
Anxiety (HADS-D)	43	6 (2–9)	26 (61)	9 (21)	8 (19)
Depression (HADS-D)	43	3 (1–6)	35 (81)	7 (16)	1 (2)
Self-assessment of distractability (FEDA questionnaire)	43	51 (40–57)	32 (74)	11 (26)	–
Self-assessment of fatigue (FEDA questionnaire)	43	32 (26–37)	30 (70)	13 (30)	–
Self-assessment of motivation (FEDA questionnaire)	43	25 (21–28)	38 (88)	5 (12)	–

In each test, impairment was classified in patients scoring below the 10th (slightly impaired) or 2nd percentile (impaired) of age-scaled norms (RWT, WMS, FEDA), or below defined cutoff scores (MMSE, CLOX, HADS). MMSE performance was classified impaired in patients scoring < 27, for the CLOX test a cutoff < 11 was used. The FAB score was scored according to age- and education norms (≥ 16th percentile: average, < 16th and ≥ 5th percentile: slightly impaired, < 5th percentile: impaired). Anxiety and depression were scored as slightly increased when ≥ 8 and increased when ≥ 11

*CLOX* clock drawing, *FAB* Frontal Assessment Battery, *FEDA* Fragebogen erlebter Defizite der Aufmerksamkeit (German Questionnaire for self-perceived deficits in attention), *HADS*-*D* Hospital Anxiety Depression Scale, *MMSE* mini-mental state, *RWT* Regensburger Verbal Fluency Test, *WMS*-*R* Wechsler Memory Scale revised

^a^Patients completing the respective test

Frontal executive impairment as evaluated with the FAB score was significantly associated with a low relative PGV in SAH patients at baseline (PGV_SAH_/PGV_REF_ < 0.69: adjusted odds ratio = 8.81, 95%-CI = 1.46–53.10, *p* = 0.018) adjusted for education years, sex, age, H&H grade and TIV. When analyzing associations of dynamic changes of PGV in SAH patients over 1 year (∆PGV) and neuropsychological outcomes, PGV decrease was significantly associated with self-perceived impaired motivation (FEDA; Wald-statistic = 6.6, *df* = 1, *p* = 0.010) after adjusting for sex and ∆TIV.

## Discussion

The main finding of the present study is that PGV is significantly lower in SAH patients, both at 16 days and 1-year follow-up as compared to age and sex matched healthy referents. The current results also suggest an association between lower PGV and impaired neuropsychological long-term outcome.

To the best of our knowledge this is the first study systematically investigating the volume of the pituitary gland in patients with SAH. Regional and global cerebral atrophy has been previously described after SAH [15–19]. Mechanisms initiating the process of diffuse brain atrophy after SAH encompass the concept of early brain injury (EBI) including brain tissue hypoxia due to the bleeding-associated increase in intracranial pressure and the reduction in cerebral blood flow, blood–brain-barrier breakdown, global cerebral edema, oxidative stress, neurohemoinflammation and excitotoxicity [2, 31, 32]. All these factors may result in early apoptotic injury also affecting the pituitary gland. Although we could not identify early disease specific predictors for low PGV, the concept of EBI may partly explain our findings. It is important to mention that we measured PGV approximately 2 weeks after the bleeding. Therefore, other factors including endogenic or even iatrogenic suppression of the pituitary gland function may have contributed to altered PGV. In specific, the anterior lobe function is controlled by feedback mechanisms of peripheral glandular tissues and the hypothalamus [33]. Besides the initial endogenic sympathetic stress response [34], iatrogenic glucocorticoid administration due to adrenergic deficiency after SAH may influence the regulation of corticotrope cells in the anterior pituitary gland [35].

We found that older age was directly associated with a lower PGV, which is consistent with previous findings [36, 37]. However, we could not identify other clinical or bleeding-associated risk factors predicting lower PGV. This is interesting and indicates that disease severity itself may not directly translate into lower PGV. In line with our findings, hypopituitarism after SAH has not been linked to initial clinical or radiographic markers, rendering predictability and early identification of these patients difficult [5, 38, 39]. It is important to mention that mainly good-grade SAH patients with a lower complication burden followed our prospective protocol of repeated MRI scanning. Moreover, we might have missed other mechanisms leading to PGV decrease based on the methods used in our study.

Overall, PGV remained constant with sustained lower PGV in SAH patients as compared to healthy referents. However, longitudinal measurements of PGV in individual patients revealed dynamic changes over the 1-year observation time. We could identify patients with PGV increase achieving almost the volume of healthy referents in the long term. This phenomenon was even more pronounced in some patients with very low baseline PGV. On the other hand, a decrease in PGV was observed in another subgroup of patients. This is in agreement with the findings of a recent study of our group suggesting a progression of widespread axonal injury 1 year after SAH in poor-grade patients (Lenhart et al., unpublished). Interestingly, we could not identify any disease specific or demographic factors associated with PGV changes over 1 year.

Low PGV may result from either cell death or diminished cell size and contribute to SAH-mediated dysfunction of hormone-secreting cells in the anterior pituitary gland. Pooled prevalence rates of hypopituitarism in the acute and subacute phase after SAH are as high as 49% [8] and decrease to 26% in the chronic phase [7, 8] with considerable variation across different studies. Clinical manifestations of hypopituitarism may resemble neuropsychological impairments, which are commonly observed after SAH. According to this hypothesis, the extent of PGV decrease was associated with worse motivation as assessed by the FEDA questionnaire in our patients. Mood disorders, anxiety, depression and fatigue substantially diminish long-term quality of life after SAH [2, 40]. Preliminary data also suggest that pituitary dysfunction and specifically growth-hormone (GH) deficiency contribute to poor quality of life, emphasizing on the need of detecting and treating modifiable causes in these patients [5, 9]. Symptoms of GH deficiency also include fatigue as well as impairment of attention and memory [4, 5]. This is of interest, since isolated GH deficit is claimed to be the most common affected axis in SAH patients [7, 8]. Due to the anatomical site with blood supply via the long hypophysial portal system, gonadotrophs and somatotrophs are most vulnerable. It is important to mention that somatotrophs are the predominant cell type of the anterior gland making up approximately 45% of pituitary cells [33]. This may explain the link between decreased volume of the pituitary gland and the association with neuropsychological impairments resembling GH deficiency. We could not find an association between baseline PGV and worse motivation at 1 year but further decrease in PGV may explain at least in some cases late-onset hypopituitarism.

We found a significant association between lower PGV at baseline and the FAB summary score assessed at 1 year after SAH. The FAB provides a useful tool to assess executive dysfunctions in several neurological conditions including Alzheimer’s disease or stroke [29, 41, 42]. As already mentioned, symptoms of GH deficiency involve executive dysfunctions such as impairment of attention and memory [4]. One might speculate that patients with low PGV exhibit some degree of pituitary dysfunction and consequently perform worse in the FAB. This hypothesis is supported by preliminary data of a pilot study conducted in TBI patients suggesting that cognitive impairments may be partially reversible with GH substitution [43]. In contrary, poor performance in the FAB may simply reflect decreased energy levels or poor motivation subsequent to hypopituitarism to complete the testing appropriately. It is very important to emphasize, that our study results do not proof causality based on the experimental design. Still our data would support a carefully designed prospective study to further elaborate on these findings.

## Limitations

There are several other limitations that merit attention. First, only patients with baseline and 1-year follow-up MRI-scan were included suggesting a trend toward good-grade patients with favorable outcome. Still, the whole spectrum including all clinical severity grades was represented and it is likely that PGV is even lower in poor-grade SAH patients with worse outcome. Second, we did not systematically measure hormone levels and can therefore not conclude an association between low PGV and pituitary dysfunction. Of all pituitary hormones, only TSH was routinely measured in all patients but was not integrated in the final statistical models as we did not find an association in univariate analysis and a selected view on pituitary hormones would have introduced a selection bias. Low PGV may even not be clinically significant since a large percentage of glandular cells needs to be destructed before clinical hypopituitarism is evident [4]. Moreover, patients were not specifically followed for various symptoms of hypopituitarism. Consequently, our study remains hypothesis generating and needs prospective confirmation. Next, the PGV is relatively small which would entail a huge bias when variability of the quantification of PGV is high. In our study, 2 independent neuroradiologists assessed PGV blinded to clinical data resulting in a high interrater reliability with an ICC of 0.979. Finally, this is a single center study and generalizability is therefore limited.

## Conclusion

The current study is the first to demonstrate significantly lower PGVs after SAH compared to healthy referents and reveals dynamic changes of PGV within 1 year. A possible association between low PGV and pituitary dysfunction as well as impaired neuropsychological long-term outcome needs further studies including neuroendocrine hormone measurements.
